# *Caenorhabditis* Intervention Testing Program: all-trans retinoic acid-related compounds tamibarotene and bakuchiol do not extend lifespan in *Caenorhabditis* nematodes

**DOI:** 10.17912/micropub.biology.001517

**Published:** 2025-02-12

**Authors:** Stephen A. Banse, Anna L. Coleman-Hulbert, Christine A. Sedore, Erik Johnson, Gordon J. Lithgow, Monica Driscoll, Patrick C. Phillips

**Affiliations:** 1 Institute of Ecology and Evolution, University of Oregon, Eugene, Oregon 97403, USA; 2 The Buck Institute for Research on Aging, Novato, California 94945, USA; 3 Department of Molecular Biology and Biochemistry, Rutgers University, Piscataway, New Jersey 08854, USA

## Abstract

The *Caenorhabditis* Intervention Testing Program recently characterized the longevity-promoting effects of the vitamin A derivative all-trans retinoic acid (atRA). Here, we test two atRA-related compounds, tamibarotene and bakuchiol, for longevity effects in three strains of *Caenorhabditis* species. Both tamibarotene, a potent RAR agonist, and bakuchiol, a meroterpene derived from *Psoralea corylifolia* , showed no significant increase in lifespan across a dosage range of six concentrations. Additionally, bakuchiol was broadly toxic at higher doses. These findings highlight the specificity of atRA's longevity effects and suggest that compounds related to atRA may not universally promote lifespan extension.

**Figure 1. Longevity of Caenorhabditis nematodes under adult drug exposure:**
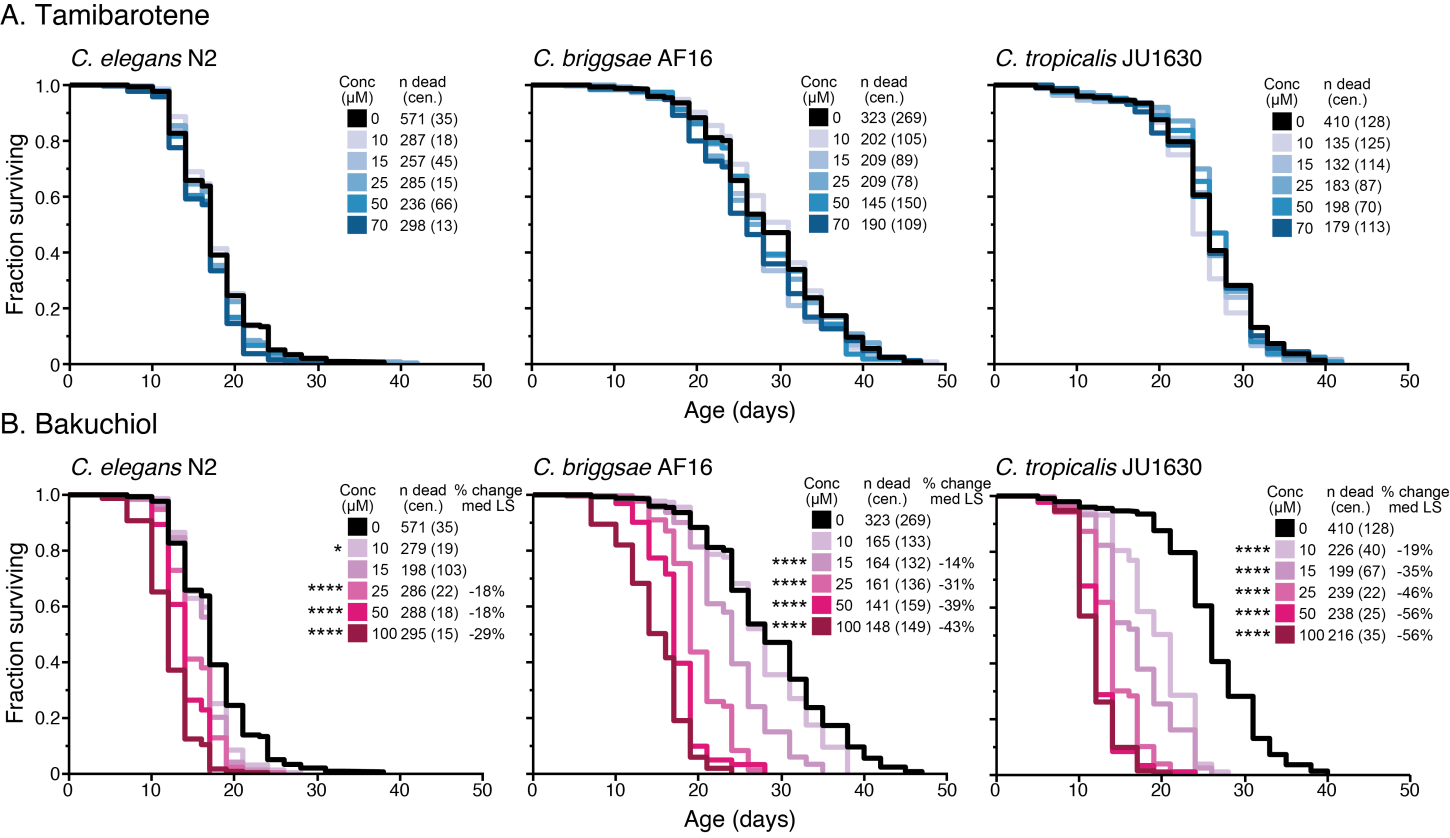
Survival curves for *C. elegans* strain N2, *C. briggsae* strain AF16, and *C. tropicalis* strain JU1630 exposed to 0, 10, 15, 25, 50, and 70 µM (A) tamibarotene or 100 µM (B) bakuchiol starting on the first day of adulthood. Statistical comparisons were made with a Cox proportional hazards (CPH) mixed-model using the coxme v.2.2-5 package in R. Asterisks represent *p* -values from the CPH model such that **** *p* <.0001, *** *p* <.001, ** *p* <.01, and * *p* <.05.

## Description

The *Caenorhabditis* Intervention Testing Program (CITP) is tasked by the NIA to characterize the lifespan effects of compound exposure across a genetically diverse test-set of *Caenorhabditis* nematodes (Lucanic et al. 2017) . The core premise of the CITP is that compounds that are efficacious across the test panel of *Caenorhabditis* strains (which includes *C. elegans* , *C. briggsae* , and *C. tropicalis* representatives) should be enriched for genetic background-independent modes of action.

CITP recently characterized the longevity-promoting retinoid all-trans retinoic acid (atRA; Banse et al. 2024b), a widely conserved retinoic acid receptor (RAR) ligand (Albalat and Cañestro 2009; Albalat 2009; Fonseca et al. 2020), which was previously shown to increase nematode lifespan (Statzer et al. 2021) . In mammals, many physiological effects of atRA are initiated through binding to RARs. In *C. elegans* , there is ample evidence for an endogenous atRA pathway (Kostrouch et al. 1995; Garofalo et al. 2003; Chen et al. 2018) , although no obvious *C. elegans* RAR homologs have been identified. Our analysis of atRA impact on *Caenorhabditis* strains (independently reproduced at three distinct laboratories following common protocols) confirmed a longevity outcome and demonstrated that atRA functions through the conserved longevity factors AKT/ AKT-1 /2, AAK-2 /AMPK, SKN-1 /Nrf2, and HSF-1 /HSF1 (Banse et al. 2024b) . With an interest in how broadly atRA pathway-related interventions might extend *Caenorhabditis* lifespan, we sought to determine if two additional atRA-related compounds, tamibarotene and bakuchiol, exert similar biological effects.

Tamibarotene is a synthetic retinoid that is a potent and selective agonist of the retinoic acid receptors RARα/β (Fukasawa et al. 1993) . In clinical use, tamibarotene has been found to be better tolerated than atRA, potentially because the RARα/β-selective retinoid does not bind or activate RARγ or the retinoid X receptors (RXRs) (Fukasawa et al. 1993) , and has reduced affinity for receptor CRABP (Jetten et al. 1987; Takagi et al. 1988) . We therefore measured tamibarotene impact on lifespan in the CITP test panel of three strains representing different species of *Caenorhabditis* nematodes ( Figure 1A ). We observed no significant effects on longevity across the tested concentration range (10 µM, 15 µM, 25 µM, 50 µM, and 70 µM). Tamibarotene may lack bioactivity in *Caenorhabditis* strains or may not be taken up efficiently by the test strains; another possibility is that, although we test a wide range of compound concentrations, our approach may have missed an effective compound dose. Overall, under the tightly controlled conditions we used, tamibarotene does not modulate *Caenorhabditis* lifespan.

As the potent RAR agonist tamibarotene failed to induce longevity, we considered other compounds that may function similarly to atRA via the non-canonical (non-RXR) modes of action of atRA. Bakuchiol is a compound found in *Psoralea corylifolia* , an herb with historic uses in Ayurvedic and Chinese medicine. Because bakuchiol has been shown to phenocopy atRA in some cases, this compound has been presented as an alternative to retinoids for dermatological phenotypes (Chaudhuri and Bojanowski 2014; Dhaliwal et al. 2019). Importantly, while bakuchiol phenocopies atRA, it does not function through the traditional RXR-like pathways; instead, bakuchiol targets mitochondrial proteins, prohibitins, and voltage-dependent anion channels (Shoji et al. 2024) . Bakuchiol and atRA induce transcriptional responses that overlap for potentially relevant gene classes, including cytochrome P450 and extracellular matrix genes (Chaudhuri and Bojanowski 2014) , p38 signaling (Lim et al. 2019) and *hsf-1* transcription (Ranjan et al. 2023) , pathways that we found are regulated by atRA in *C. elegans* (Banse et al. 2024b) . We therefore measured longevity of the representative CITP test strains under bakuchiol exposure ( Figure 1B ). Despite overlap in target pathways with atRA, we found that bakuchiol either had no effect or was toxic, shortening *Caenorhabditis* lifespan. Taken together, our data support that atRA may engage longevity-promoting retinoic acid signaling pathways to an optimal level that is not readily attained by other pathway modulators.

## Methods

We assayed lifespan in response to compound exposure in three *Caenorhabditis* species using our previously published workflow (Banse et al. 2024a) . In brief, for two separate biological replicates, animals were age-synchronized by timed egg-lays on standard 60 mm diameter Nematode Growth Media (NGM) plates and transferred at a density of 50 individuals per 35 mm treated plate in triplicate when they reached day one of adulthood (for control plates, there were six replicates total of 50 animals each). Bakuchiol and tamibarotene were dissolved in DMSO and diluted appropriately such that addition of 132.5 µl of solution to 35 mm diameter plates containing NGM with lawns of *E. coli* OP50-1 and 51 µm FUdR would generate the following final compound concentrations: 0 µM (control), 10 µM, 15 µM, 25 µM, 50 µM, and 70 µM (tamibarotene) or 100 µM (bakuchiol). The final concentration of DMSO in all plates was 0.25%. Animals were maintained at 20°C and 80% RH, moved to fresh plates on the first, second, and fourth ( *C. tropicalis* ) or fifth ( *C. elegans* and *C. briggsae* ) day of adulthood and then once weekly afterward. Thrice weekly, animals were scored for movement after prodding with a 0.2 mm diameter platinum wire and death was considered a lack of movement.

We performed statistical analyses as previously described (Lucanic et al. 2017) . Briefly, survival was analyzed both with a generalized linear model (lme4 package v1.1-35), and a mixed-model Cox-Proportional Hazards (CPH) model (coxme package v2.2-22; Therneau 2020) using the R statistical language (R Core Team 2021; v4.3.3). The effect of compound treatment was tested using CPH analysis within each strain to allow for each compound treatment replicate to be compared to its specific control (multcomp package v1.4-26). All published CITP data can be accessed on the CITP Data Portal (citpaging.org/portal, v2.1), and on figshare.com along with the R scripts used for analysis (DOI: 10.6084/m9.figshare.c.7628138).

## Reagents

We conducted experiments using *C. elegans* N2_PD1073 (Banse et al. 2019; Yoshimura et al. 2019) , *C. briggsae* AF16 (Golden et al. 1983) , and *C. tropicalis* JU1630 (Teterina et al. 2022) . N2_PD1073 and AF16 are available from the CGC, which is funded by NIH Office of Research Infrastructure Programs (P40 OD010440) and *Caenorhabditis* Natural Diversity Resource (Cook et al. 2017) , while JU1630 is available from CITP. For chemical interventions, tamibarotene (AmBeed A197861) and bakuchiol (AmBeed A182762) were obtained in solid form and dissolved in DMSO (Sigma-Aldrich).
